# A videoconferencing group-based psychoeducation for family caregivers of people living with dementia: a feasibility study

**DOI:** 10.1186/s12877-026-07010-y

**Published:** 2026-02-09

**Authors:** Jackie Hoi Man Chan, Ken Hok Man Ho, Helen Yue Lai Chan

**Affiliations:** 1 The Nethersole School of Nursing, Faculty of Medicine, The Chinese University of Hong Kong, 6-8/F, Esther Lee Building, Shatin, Hong Kong SAR China; 2 Department of Nursing, Faculty of Health Sciences, Higher Colleges of Technology, Dubai, United Arab Emirates; 3 School of Nursing and Midwifery, La Trobe University, Melbourne, Australia

**Keywords:** Dementia, Family caregivers, Psychoeducation, Psychosocial intervention, Feasibility, Acceptability, Caregiving self-efficacy, Simulation

## Abstract

**Background:**

The family caregivers of people living with dementia may be limited to staying at home because of their engagement in caregiving tasks, living remotely and difficulty seeking substitute care. Therefore, family caregivers may be unable to attend conventional in-person group-based psychoeducation, which is important in enhancing their caregiving self-efficacy through informational and emotional support. An alternative mode of delivering psychoeducation remotely, such as tele-technology, may better facilitate family caregivers’ participation in psychoeducation.

**Methods:**

A quasi-experimental study was conducted to test the feasibility, acceptability, and helpfulness (with preliminary effect observed with RSCSE score) of videoconferencing group-based psychoeducation among family caregivers of people with early-to-moderate-stage dementia. Adult family caregivers of community-dwelling persons with mild-to-moderate-stage dementia were recruited. They received the intervention either online via Zoom videoconferencing or in-person for six weeks. Feasibility was measured in subject recruitment, attrition upon intervention completion and completion rate. Acceptability was assessed based on participants’ satisfaction, and qualitative interviews were used for process evaluation. The preliminary effect on caregiving self-efficacy was measured by the Revised Scale of Caregiving Self-Efficacy (RSCSE) at baseline and immediately after the intervention. The Mann‒Whitney U test was performed to examine changes in the mean RSCSE score between groups postintervention.

**Results:**

Sixty subjects were recruited from two community centers for older adults and Facebook, which yielded a recruitment rate of 78%. The attrition rate upon intervention completion was 8.3%. The completion rates were 96.5% and 93.5% in the in-person and online groups, respectively. The intervention was acceptable, with a mean satisfaction score of 4.7 out of 5 in the online group and 4.5 in the in-person group. The qualitative findings revealed four themes: advantages of the online format, active learning, perceived benefits and implementation challenges. Family caregivers in the online group reported a significantly greater improvement in the responding to disruptive behaviours subscale of the RSCSE post intervention (*r* = 0.26; *p* = 0.037).

**Conclusions:**

The newly developed videoconferencing group-based psychoeducation was feasible and acceptable, and the preliminary effects of the online format were comparable with those of the in-person intervention. A larger robust study is needed to examine the intervention effects.

**Trial registration:**

This study was retrospectively registered at ClinicalTrials.gov (Identifier: NCT06042634).

**Supplementary Information:**

The online version contains supplementary material available at 10.1186/s12877-026-07010-y.

## Introduction

The people living with dementia (PLWD) experience progressive cognitive and functional decline, resulting in high dependency and therefore negative physical and psychosocial consequences in their overburdened family caregivers [1]. Caregiving self-efficacy, a perceived confidence in caregiving [2], effectively reduces caregiver burden and depressive symptoms [3–5] and promotes the physical health [6] of family caregivers of PLWD. The World Health Organisation [7] recommended enhancing the caregiving self-efficacy of family caregivers of PLWD through informational (e.g., basic dementia disease knowledge and communication skills) and emotional support (e.g., stress coping strategies) in psychoeducation. Improving the knowledge and skills of caregivers of PLWD in a contemporary ageing population is crucial [8]. However, family caregivers of PLWD reported barriers in attending in-person group-based psychoeducation programmes, including intense caregiving schedules, employment, finding reliable substitute care, long travel times [9–11] as well as social distancing and home confinement due to crises such as the COVID-19 pandemic [12, 13]. Therefore, alternative modes of delivering psychoeducation remotely, such as tele-technology, may better facilitate the participation of caregivers of PLWD in psychoeducation than in the in-person channel.

## Background

Enhancement in caregiving self-efficacy among family caregivers of PLWD was demonstrated in in-person group-based psychoeducation [14] and those with didactic teaching and active participation components [15]. These designs conform to self-efficacy theory by addressing the four sources of self-efficacy: (1) verbal persuasion, e.g., provision of verbal information on dementia caregiving; (2) mastery experience, e.g., hands-on training on communication skills and the management of distressed behaviours through role play; (3) vicarious experience, e.g., group discussion of caregiving experiences; and (4) physical and psychological states, e.g., practice sessions of relaxation techniques to help destress [16]. Didactic teaching of caregiving knowledge reinforces confidence in caregiving and therefore addresses verbal persuasion [17]; active participation provides skill training opportunities and therefore addresses mastery experiences [18]; group sharing of caregiving experiences allows peer learning and therefore addresses vicarious learning [14]; the physical and psychological state is addressed by practicing relaxation techniques in active participation [19].

Studies have suggested that family caregivers of PLWD can access videoconferencing group-based psychoeducation, which has demonstrated effectiveness in enhancing their caregiving self-efficacy [18, 20]. Videoconferencing group-based psychoeducation simulates in-person group-based psychoeducation through synchronous virtual group meetings [18, 21, 22]. Therefore, videoconferencing group-based psychoeducation allows real-time interaction with other caregivers and facilitators, wherein peer learning, didactic teaching, active participation, and psychological support could be provided in a similar way as in-person group-based psychoeducation does [18, 21, 22]. Videoconferencing group-based psychoeducation may be an alternative to in-person group-based psychoeducation, especially for those living in rural areas who need to access support remotely. This may have significant implications for service provision for those family caregivers of PLWD who cannot attend in-person group-based programmes. Therefore, studies comparing in-person group-based psychoeducation versus videoconferencing group-based psychoeducation are necessary to demonstrate whether the two modes of psychoeducation are comparable in enhancing caregiving self-efficacy. However, there is a dearth of information comparing psychoeducation in the two modes, which represents a research gap for future studies.

Furthermore, feasibility issues related to videoconferencing group-based psychoeducation, which are the uncertainties to address before proceeding to a future definite trial, have been reported [23]. The feasibility issues reported were high attrition (25%) due to scheduling difficulties [18]; fair recruitment (64.7%) with conventional recruitment strategies (printed flyers and mailing of study information) [22]; overwhelming technology [22, 24]; and unstable internet connectivity in rural areas [22]. Although internet utilization is high in Hong Kong (99.4% via smartphones; 83.3% via personal computers) [25], the digital literacy, an ability to employ digital devices to find, evaluate and use information [26], of the local family caregivers of PLWD is unknown. Therefore, whether the local family caregivers of PLWD are able to access and adopt caregiving information delivered through videoconferencing group-based psychoeducation is an issue worthy of concern.

Literature suggests that family caregivers had a lack of knowledge and skill related to dementia caregiving, particularly in managing behavioural and psychiatric symptoms of dementia (BPSD), handling their own negative emotions and thoughts related to caregiving, and finding respite care [27]. These essential dementia caregiving knowledge and skills are the cornerstones of caregiving self-efficacy [2], which provides a basis for the development of a psychoeducational programme in the current study. Our proposed videoconferencing group-based psychoeducation for family caregivers of PLWD was informed by the self-efficacy theory [16]. The intervention encompasses four sources of self-efficacy, including verbal persuasion, mastery experience, vicarious experience, and physical and psychological states. This study was a feasibility study, the results of which were used to inform a subsequent definite trial. The objectives of this feasibility study were: (1) to develop the interventions encompassing four sources of self-efficacy for caregivers in order to increase caregiving self-efficacy, and (2) to test its feasibility, acceptability, and helpfulness (with preliminary effect observed with RSCSE score).

## Methods

### Hypotheses

The current study hypothesised that, upon completion of the intervention, the family caregivers in the intervention group, who received videoconferencing group-based psychoeducation, would report a significantly greater improvement in caregiving self-efficacy than those in the control group, who received in-person group-based psychoeducation.

### Study design

This feasibility study employed a quasi-experimental pretest‒posttest design to compare videoconferencing group-based psychoeducation with in-person group-based psychoeducation. Since the study was conducted from July 2022 to February 2023 when social distancing policy was enforced during the COVID-19 pandemic, in-person group-based psychoeducation for the control group was withheld for the first 5 months of study. The study reported here was in compliance with the Declaration of Helsinki and was retrospectively registered on 18/09/2023 at clinicaltrials.gov (Identifier: NCT06042634). The Consolidated Standards of Reporting Trials (CONSORT) guidelines for pilot and feasibility trials were used for reporting this study [28].

### Study settings

The study was conducted in collaboration with two District Elderly Community Centers (DECCs) in Hong Kong. DECCs are funded by the Hong Kong government. DECCs provide community support services to older adults at the district level, thereby enabling them to remain in the community. The in-person group-based psychoeducation was conducted in the DECCs. The videoconferencing group-based psychoeducation was conducted via Zoom, which is an online platform for synchronous virtual video or audio-only meetings. The videoconferencing service provided by Zoom is free of charge and is password protected.

### Participants

Eligible participants were Cantonese-speaking adults who were (i) aged 18 years or above; (ii) cognitively competent (i.e., Mini Mental State Examination ≥ 23) [29]; (iii) taking care of a family member diagnosed with dementia of mild to moderate stage and required assistance in physical activities of daily living (ADL) (i.e., score ≥ 2 on the Activity of Daily Living scale [2, 30]; (iv) providing at least 5 h of caregiving per week in the past month [31]; and (v) had low caregiving self-efficacy (i.e., score ≤ 3 on the Caregiving Competence Scale [10, 32]). Caregivers of PLWD were excluded if they (i) were participating in any other psychosocial interventions; (ii) had psychiatric illnesses and having active treatment; or (iii) did not have access to the internet on any type of electronic device such as a smartphone or laptop.

### Recruitment

Caregivers of PLWD were recruited from two sources: two DECCs and Facebook. The two DECCs were referred by a psychogeriatric nurse who served as the reviewer of the intervention fidelity of the current study. Printed flyers were physically displayed in the DECCs, and advertisements were posted on Facebook. Interested participants approached the first author through telephone and were screened for eligibility. Informed consent was collected physically at the DECCs or via Google Forms.

### Sample size

The feasibility study required 30 participants per arm to test the intervention before a future definitive trial [33]. Therefore, with two groups in this study, the total sample size was set at 60.

### Intervention

Table 1 shows an overview of the videoconferencing group-based psychoeducation programme. Guided by self-efficacy theory, a manualized psychoeducation was developed based on critical literature reviews: (1) systematic review of 8 RCTs of online psychoeducation to identify the content and modality of the current intervention, and (2) scoping review of 29 psychoeducational programmes to identify the strategies in promoting caregivers’ active participation in psychoeducation [19]. The six topics covered in the current programmes entailed essential knowledge and skills in dementia family caregiving, which aimed to enhance the caregiving self-efficacy among the family caregivers of PLWD.

**Table 1:** Overview of the psychoeducation programme

Weekly topic	Content			
	Didactic teaching	Discussion topic	Assignment topic	Simulation scenario
1. Understanding dementia	Introduce the nature of dementia, stages of dementia and related care needs.	Current challenges in caregiving and the way to manage these challenges	Identify expectations in caregiving	Not applicable
2. Stress and coping	Introduce signs of stress, negative emotions related to caregiving and ways to deal with negative emotions and stress.	Negative feelings towards caregiving and own methods in dealing with caregiving stress	Record negative feeling every day and how to manage for the week	Not applicable
3. Communication skill	Introduce verbal and non-verbal communication skills and strategies for effective communication.	Communication skills used before and if they worked	Identify and record the effective communication skills used for the week	Asking for preferences and learning to say no to unreasonable demands
4. Daily care in dementia caregiving	Introduce daily schedule and social activities planning, strategies for promoting nutrition and daily care, and strategies in disease prevention.	Social activities joined with your loved one before	Plan the social activities for your loved one for the next week.	Dealing with refusal to eating and bathing
5. Behavioral and psychological symptoms of dementia (BPSD) and its management	Introduce common BPSD and strategies in managing common BPSD	The experience in managing BPSD	Identify the BPSD and record your management for the week.	Dealing with hallucination with delusion
6. Community resources and future care planning	Introduce community resources related to dementia, future care planning. Review communication skill and strategies in managing BPSD.	The community resources used	Plan the future care for the loved one	Dealing with wandering behavior and angry outbursts

The programme included two core components: didactic teaching and active caregiver participation. The didactic teaching provided information support about dementia caregiving which was delivered via PowerPoint presentations. Active caregiver participation allowed the participants to learn in active ways. Four teaching and learning methods for active participation, that could fulfil the sources of self-efficacy, were employed: simulation and written assignments for hands-on skill training, group discussion for peer sharing of caregiving experiences, and practicing sessions of relaxation techniques to address negative emotions [19]. The participants went through six weekly psychoeducation sessions in a small group of five to eight. Each psychoeducation session lasted for 120 min.

Moreover, strategies enhancing active caregiver participation were incorporated [19]. The topics of discussion and written assignment were tailored to the experiences of the caregivers. The interventionist facilitated peer sharing and support by setting clear ground rules, including confidentiality and mutual respect, at the beginning of the discussion. In addition, the interventionist collaborated with caregivers by maintaining good interpersonal skills, including being approachable, encouraging, non-judgmental and sensitive to the needs and feelings of caregivers.

Furthermore, simulated patients, underpinned by the experiential learning theory [34], were employed to provide authentic conversations and facial expressions when roleplaying simulation scenarios around common communication challenges and behavioural and psychological symptoms of dementia (BPSD). First, the participants received concrete learning when practicing communication skills and management of BPSD in roleplay. They were expected to verbally respond to the simulated patient thoughtfully, which helped to build mastery experience. In each round of simulation, one participant enacted with the simulated patient while the others observed it, which contributed to vicarious experience. The participants were then debriefed to reflect on what was done effectively and areas for improvement. Next, the caregivers were facilitated to conceptualise and generalise the learning to various caregiving situations. The interventionist also provided feedback to reinforce participants’ confidence in caregiving, which contributed to verbal persuasion. Finally, the caregivers were encouraged to apply the skills learned between psychoeducation sessions, which were reviewed at the next session. Because the simulation focused on authentic conversation, it could be delivered either in face-to-face or videoconference formats. Each scenario was limited to 5 min and run twice.

Weekly home assignments about applying the learned knowledge in the caregiving situation, which would be reviewed in the next session, were given to the participants. Before each session ended, the interventionist guided the participants to practice deep breathing to reduce stress and promote relaxation [35]. An educational booklet was developed that covered the content of didactic teaching, discussion topics, simulation scenarios, home assignments and deep breathing instructions. The booklet, which was distributed to caregivers in centers or mailed by post, helped enhance knowledge acquisition during psychoeducation sessions and at home.

To enhance intervention fidelity, the intervention for both the online and in-person groups was delivered by the first author, who is a registered nurse with experience in dementia care and simulation with family caregivers. In addition, a 3-hour training was given to the simulated patients to ensure standardized performance in the simulation, wherein understanding the scenario background and responding to participants verbally were included. Two female simulated patients were recruited. They were healthy individuals who were over 60 years old, and had no relationship with the participants. Furthermore, two psychogeriatric nurses reviewed and rated one-third of the video recordings of psychoeducation sessions on a 5-point Likert scale according to a fidelity checklist that consisted of 4 items: the interventionist’s adherence to the intervention protocol, the dosage of the intervention delivered, the simulated patients’ adherence to the simulation scenarios and the responsiveness of the participants. The mean intervention fidelity calculated was 4.7/5 in both groups.

### Control condition

Participants in the control group received in-person group-based psychoeducation, which had the same content and flow of presentation as videoconferencing group-based psychoeducation. The intervention and control groups differed only in terms of mode of delivery. The in-person group-based psychoeducation was presented physically in the DECCs.

### Outcome and measures

Sociodemographic data of the participants, including age, gender, relationship with the care recipient, level of education, and the caregiving experience of the participants were collected. Additionally, sociodemographic data of the care recipient, including age, number of months since diagnosis, their Montreal Cognitive Assessment (MoCA) score, and their score of Activity of Daily Living, were collected.

Feasibility was determined in both the intervention and control groups, and was evaluated by recruitment, attrition upon intervention completion, and completion rate. The recruitment rate was calculated as the proportion of eligible subjects who consented to join the study. The attrition rate was calculated as the proportion of consented subjects who dropped out of the study. The reasons for attrition, such as refusal, were recorded. The completion rate of participants was calculated as the proportion of participants who attended at least four out of six sessions [10]. This feasibility criterion was set at 80% [35]. Class attendance was checked by the interventionist, and reasons for absenteeism were documented. The appropriateness of the outcome measures was evaluated by asking about participants’ perceived difficulty in completing questionnaires and examining the missing data in each measure. The completion rate of the study instrument was calculated as the proportion of data completed by the consenting participants.

Acceptability was determined in both intervention and control groups, and was evaluated by a self-developed 6-item satisfaction questionnaire to provide ratings on the content, format and appropriateness of the intervention. The items were rated on a 5-point Likert scale (1 = strongly disagree, 2 = disagree, 3 = no comment, 4 = agree, 5 = strongly agree). A higher total score represented greater satisfaction with the intervention. The acceptability criterion was set at 4 out of 5 of the average satisfaction scores [36]. This study also included a qualitative study as a process evaluation to explore the participants’ experiences with the videoconferencing group-based psychoeducation. Individual semi-structured interviews were conducted by a research assistant via Zoom video conferencing. Participants from the online group, who attended at least 4 psychoeducation sessions, were recruited and asked for their experience with the programme. A semi-structured interview guide was used to guide the interviews. Table 2 presents the guiding questions.

**Table 2:** Guiding questions of semi-structured interview

Interview guide for participants
How was your experience of the intervention program?
How did you feel when you participated in simulation, group discussion, written assignment and relaxation exercises?
What did you gain from the simulation, group discussion, written assignment and relaxation exercises?
To what extend was this program useful to you?

The Chinese version of the Revised Scale of Caregiving Self-Efficacy (RSCSE) [37] was used to measure the caregiving self-efficacy of caregivers. The RSCSE has three subscales: (i) self-efficacy in obtaining respite; (ii) self-efficacy in responding to disturbed behaviors; and (iii) self-efficacy in cutting upsetting thoughts. Each subscale contains five items totaling 15 items. The rating scale for each item ranges from 0 (Cannot do at all) to 100 (Certainly can do). The score of each item within the subscale are summed to provide a subscale score. The higher the score is, the greater self-efficacy in that caregiving domain. It has been validated in Hong Kong, and the Cronbach’s alpha coefficients for the three subscales in the original and Chinese versions are 0.89 or above [2, 37]. The Cronbach’s alpha coefficients for this study was 0.70.

### Group allocation

Participants were allocated to the group according to the time they enrolled to the study. Since face-to-face interaction was limited by social distancing policy in the first 5 months of the study period, subject recruitment was only for intervention group. Subject recruitment for the control group started from December 2022 when the social distancing policy was relaxed, and recruitment from the DECCs was resumed. The participants were informed of the group allocation at the time of recruitment.

### Data collection

The sociodemographic data and outcomes were collected via telephone by a research assistant who was blinded to the group allocation. Interview was conducted by the research assistant through videoconferencing. The study instrument was administered to all the participants at baseline (T0) and immediately after the intervention (T1). All participants were also asked to complete the satisfaction questionnaire at T1. Questions for the satisfaction questionnaire and the RSCSE were read to the participants. Individual semi-structured interview was conducted in the intervention group by a research assistant via videoconferencing. Each participant received an HK$100 cash voucher upon completion of the study to appreciate their time spent and compensate for the cost of travel to the study sites.

### Data analysis

Quantitative data were analysed via IBM SPSS Statistics version 26.0. Descriptive statistics were used to summarize the demographic data and feasibility outcomes, including the recruitment rate, attrition rate, completion rate of the intervention, participants’ satisfaction score and completion rate of the instrument. The chi-square test for categorical variables and the Mann‒Whitney U test for continuous variables were used to examine the homogeneity of the participants in the two groups. Within-group differences in the mean RSCSE score were tested via the Wilcoxon signed-rank test. Between-group differences in the mean RSCSE score post-intervention were tested via the Mann‒Whitney U test. The effect size was approximately calculated by dividing the z value by the square root of the total number of subjects [38] and was reported as Cohen’s r value [39]. The cut-off points of small, medium and large effect sizes were 0.1, 0.3 and 0.5, respectively. All analyses were considered significant at *p* ≤ 0.05 (2-tailed).

The audio-recorded interviews were transcribed verbatim in Chinese by the first author for qualitative content analysis [40]. The transcripts were managed with the support of NVivo 14 for systematic organization of the data. The transcripts were checked line by line against the audiotape to ensure that the transcription process was accurate and no personal ideas of the researcher were incorporated into the study. To ensure the trustworthiness of data analysis, prolonged engagement and audit trail were employed [41]. Two researchers (the first and second authors) participated in the data analysis process and met regularly to discuss issues regarding the analysis process. They read the transcripts multiple times to become familiar with the data and then coded the data individually, which were then compared, and any discordant coding was resolved through discussion. Then, they developed initial themes and sub-themes together. Critical discussion and revision of the themes and subthemes were carried out among the research team until a consensus was reached. This ensured the codes, themes and sub-themes developed were more accurate and objective [41]. Additionally, an audit trail, which documented the data collection, data analysis process, and the characteristics of the informants, was kept. This allowed readers to evaluate if the findings could be applied to another setting [41].

Illustrative quotes were selected through critical discussion between the authors. The first author and second author then translated the themes, subthemes and quotes into English independently, which were compared and adjusted until an agreement was reached for the final English version. The third author back-translated the English version into Chinese. Discrepancies between the Chinese and English languages were reviewed and discussed among the research team until an agreement on meaning was reached [42].

### Ethical considerations

This study complied with the Declaration of Helsinki and was approved by the Joint Chinese University of Hong Kong-New Territories East Cluster Clinical Research Ethics Committee (CREC Ref. No. 2022.248). Participation was voluntary, and the participants could withdraw from the study at any time without any consequences.

## Results

### Study participants

Table 3 shows the sociodemographic characteristics of the participants. The mean age of the 60 participants was 59.2 years (SD = 12.6; range from 30 to 93). The majority of them were female (83%), child of (55%), lived with the care recipient (91.7%), were not working (60%), and had an education level secondary to tertiary (76%). The mean caregiving time per week was 71.4 h (SD = 59.6), and the mean caregiving experience was 28.4 months (SD = 23.2). The intervention and control groups did not differ in the score of caregiving self-efficacy, and the mean score was 55.6 (SD = 14.8). The mean age of the care recipients was 79.2 years (SD = 8.9; range from 56 to 99). The mean number of months since diagnosis was 39.5 (SD = 29.3), the mean MoCA score was 12.2 (SD = 3.6), and the mean score of Activity of Daily Living was 2.8 (SD = 1.0). Compared with those in the in-person group, the participants in the online group were significantly younger (*p* < 0.001), adult children (*p* < 0.001), were working (*p* = 0.008) and had higher education levels (*p* < 0.001). Care recipients in the online group were significantly older (*p* = 0.021) and had lower MoCA scores (*p* < 0.001).

**Table 3:** Demographic characteristics of the participants in the online group and in-person group

	ALL	Online group	In-person group	*p*
Variables	(*n* = 60)	(*n* = 30)	(*n* = 30)	
Caregiver				
Mean age (SD)	59.2 (12.6)	50.7 (8.8) (range 30–74)	67.8 (9.7) (range 48–93)	< 0.001
Mean caring experience (months) (SD)	28.4 (23.2)	28.3 (26.8)	28.6 (19.5)	0.150
Mean caregiving time per week (SD)	71.4 (59.6)	56.4 (49.2)	86.3 (65.8)	0.052
Sex, n (% female)	50 (83.3)	27 (90.0)	23 (76.7)	0.166
Marital Status, n (%)				0.028
Married	47 (78.3)	20 (66.7)	27 (90.0)	
Single	13 (21.7)	10 (33.3)	3 (10.0)	
Education Level, n (%)				< 0.001
Primary	14 (23.3)	1 (3.3)	13 (43.3)	
Secondary	23 (38.3)	10 (33.3)	13 (43.3)	
Tertiary	23 (38.3)	19 (63.3)	4 (13.3)	
Relationship with PLWD, n (%)				< 0.001
Spouse	20 (33.3)	2 (6.7)	18 (60.0)	
Child	33 (55.0)	26 (86.7)	7 (23.3)	
Sibling	4 (6.7)	1 (3.3)	3 (10.0)	
Parent	2 (3.3)	0 (0.0)	2 (6.7)	
Grandchild	1 (1.7)	1 (3.3)	0 (0.00)	
Living with care recipient, n (%)				
Yes	55 (91.7)	26 (83.8)	29 (96.6)	0.161
Working status, n (%)				0.008
Non-working	36 (60.0)	13 (56.7)	23 (76.7)	
Working (full time and part-time)	24 (40.0)	17 (43.3)	7 (23.3)	
RSCSE score, mean (SD)				
Obtaining Respite	47.52 (30.1)	47.43 (30.2)	47.78 (30.1)	0.826
Responding to disruptive behavior	63.4 (20.4)	65.33 (20.4)	60.18 (21.4)	0.457
Controlling upsetting thoughts	54.1 (20.0)	52.67 (20.81)	54.83 (20.28)	0.520
Care recipient				
Age, mean (SD)	79.2 (8.9)	81.9 (8.6) (range 56–99)	76.6 (8.6) (range 62–94)	0.021
Number of months since diagnosis, mean (SD)	39.5 (29.3)	44.3 (35.3)	34.8 (21.5)	0.210
Sex, n (% female)	37 (61.7)	24 (80.0)	13 (43.3)	0.003
MoCA score, mean (SD)	12.2 (3.6)	10.0 (3.1) (range 6–16)	14.5 (2.6) (range 9–17)	< 0.001
ADL score, mean (SD)	2.8 (1.0)	3.0 (1.1)	2.6 (0.8)	0.105

*ADL* Activity daily living, *MoCA* Montreal Cognitive Assessment, *PLWD* People living with dementia, *RSCSE* Revised Scale of Caregiving Self-Efficacy, *SD* Standard deviation

### Feasibility of the programme

The recruitment rate was 78%. Subject recruitment spanned from July to December 2022 until the sample size was reached. Two community centers referred 35 potential subjects, and another 57 potential subjects expressed interest through Facebook. Among the 92 potential participants, 16 were excluded because they were currently participating in other studies (*n* = 1), were illiterate (*n* = 1), had no medical diagnosis of dementia (*n* = 5), were care recipients residing in long-term care facilities (*n* = 5) or non-family caregivers (*n* = 4). Another 16 subjects refused to provide consent because of scheduling conflicts.

**Fig. 1:**
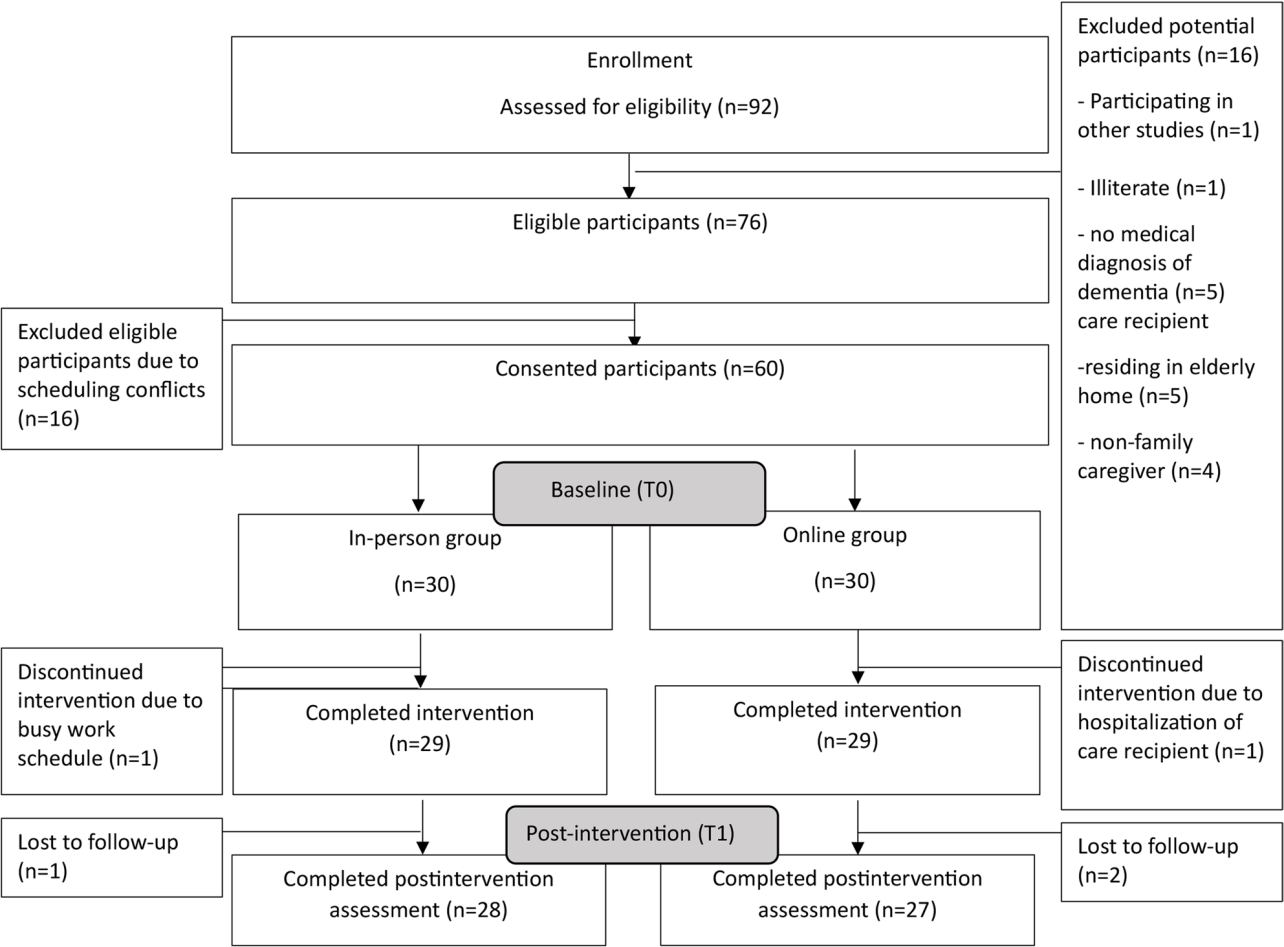
Flow diagram of the study

The overall attrition rate upon intervention completion was 8.3%. The reasons for drop-off were hospitalization of care recipients, busy work schedules and loss to follow-up. Figure 1 shows the flow diagram of the study. For the in-person class, 96.5% of the participants attended at least 4/6 sessions, and 58.6% of the participants attended 6 sessions. For the online class, 93.5% of the participants attended at least 4/6 sessions, and 61.2% of the participants attended 6 sessions. The reasons for the absence of both groups were engagement in looking after care recipients, personal matters and the hospitalization of care recipients. The completion rates for the online and in-person interventions were 93.5% and 96.5%, respectively. The completion rate of the study instrument was 95%. The participants were able to complete the RSCSE in both online and in-person groups. Two participants in the online group and one participant in the in-person group did not respond to the post-intervention measurement.

### Acceptability of the intervention

The overall satisfaction score was 4.7 out of 5 in the online group and 4.5 in the in-person group. Over 96% of the participants agreed or strongly agreed that the program provided useful information or strategies regarding caregiving in both groups. The participants were particularly satisfied with the intervention content, presentation of content and simulation (4.7/5).

### Helpfulness of the intervention

Table 4 presents within-groups comparison of mean RSCSE scores between pre- and postassessments. Both the online and in-person groups demonstrated significant enhancement in the scores of the three subscales of the RSCSE following participation in the interventions. Compared with the face-to-face group (*n* = 28), the online group (*n* = 27) demonstrated a significantly higher score in the responding to disruptive behaviors subscale post intervention, with a medium effect size (*p* = 0.037; *r* = 0.26). No significant differences were demonstrated in the scores of the obtaining respite and controlling upsetting thoughts subscales between the two groups post intervention. Table 5 presents the differences in the mean RSCSE scores between the groups post intervention.

**Table 4:** Within groups comparison of RSCSE scores between two repeated assessments

Outcome	Baseline,mean (SD)	Post-intervention, mean (SD)	Mean difference	z	p
RSCSE					
OR					
Online	47.43 (30.22)	61.46 (22.01)	14.03 (21.24)	-2.87	0.004
In-person	47.78 (30.12)	59.00 (25.26)	11.21 (18.47)	-2.77	0.006
RDB					
Online	65.33 (20.46)	76.44 (14.43)	11.11 (17.57)	-3.42	0.001
In-person	60.18 (21.47)	67.78 (15.51)	7.60 (18.65)	-1.93	0.052
CUT					
Online	52.67 (20.81)	64.53 (16.60)	11.85 (23.94)	-2.74	0.006
In-person	54.83 (20.28)	63.93 (19.44)	9.09 (21.85)	-1.83	0.066

*CUT* Controlling upsetting thoughts, *OR* Obtaining Respite, *RDB* Responding to disruptive behaviors, *RSCSE* Revised Scale of Caregiving Self-Efficacy, *SD* Standard deviation

**Table 5:** The differences in the RSCSE scores between groups post-intervention

Outcome	Online group (n=27), mean (SD)			In-person group (n=28), mean (SD)			z	p
	Baseline	Post-intervention	Mean difference	Baseline	Post- intervention	Mean difference		
RSCSE								
OR	47.43 (30.22)	61.46 (22.01)	14.03 (21.24)	47.78 (30.12)	59.00 (25.26)	11.21 (18.47)	-0.33	0.736
RDB	65.33 (20.46)	76.44 (14.43)	11.11 (17.57)	60.18 (21.47)	67.78 (15.51)	7.60 (18.65)	-2.08	0.037
CUT	52.67 (20.81)	64.53 (16.60)	11.85 (23.94)	54.83 (20.28)	63.93 (19.44)	9.09 (21.85)	-0.09	0.926

*CUT* Controlling upsetting thoughts, *OR* Obtaining Respite, *RDB* Responding to disruptive behaviours, *RSCSE* Revised Scale Caregiving Self-Efficacy, *SD* Standard deviation

### Individual semi-structured interview

Qualitative interviews were conducted individually with 27 participants who completed at least 4 sessions of online psychoeducation after the completion of the intervention. Four themes were identified: (i) advantages of the online format; (ii) active learning; (iii) perceived benefits; and (iv) implementation challenges. Subthemes were further identified within each theme and are shown in Table 6.

**Table 6:** Qualitative findings

Themes	Sub-themes
Advantages of online format	Online format was handy
	Online format facilitated open discussion
Active learning	Engaged in learning
	Listend and observed other caregivers
	Reflected on own caregiving approach
	Interacted with the facilitator
Perceived benefits	Enhanced skill in managing distressed behavior and communication
	Enhanced caregiving knowledge
	Reduced stress
Implementation challenges	Mixed feelings about simulation
	Incompletion of home assignment

#### Theme 1: advantages of the online format

The participants appreciated that ‘online format was handy’ and ‘facilitated open discussion’, which became the two sub-themes under this theme. They found that remote access allowed them to attend the intervention even though they were busy with caregiving, leading to scheduling conflicts for in-person classes. Additionally, they could save on travel when attending online classes as they live in remote areas. Moreover, the participants appreciated that the virtual platform facilitated them to discuss their own caregiving experience. They felt less embarrassed to openly express their feelings about caregiving and share both successful and unsuccessful caregiving tips.

*There is a difficulty about attending on time…caregiving occupies all my time. It wasn’t easy for us to come and attend classes as we live in the countryside*.

*I feel less embarrassed talking about my mum behind the screen…if I see them in real…I would talk less*.

#### Theme 2: active learning

The participants highlighted active learning during class and at home. They ‘engaged in learning’, ‘listened and observed other caregivers’, ‘reflected on their own caregiving approach’ and ‘interacted with the facilitator’, which became the four sub-themes under this theme. The participants appreciated engaging in simulation and home assignments. The conversation with the simulated patients provided high fidelity to the participants so that they could practice communication skills and handle distressed behaviour through verbally responding to the simulated patient. While at home, they continuously learnt through completing written assignments, which reminded them the weekly learning focus to apply the knowledge learned and evaluate their own methods.

*The actor acted very lively and made me feel immersed. The conversation is spontaneous as in real life situation…I have to react quickly and calmly with logical thinking….*

*The home assignment was about planning daily schedule of the care recipient…I would think what activities to arrange in everyday schedule and observe if he has special emotions…then I will pay more attention to these…then I will think about the triggers and how to help*.

Additionally, the participants appreciated learning from peers through listening to others’ caregiving experiences in group discussions. It helped prepare psychologically and equip with solutions for handling caregiving problems. The participants also highlighted the importance of observing how other caregivers handled a problem during the simulation. They contemplated caregiving solutions, picturing themselves going through the scenario as they watched the scene.

*There are experience sharing from other caregivers…this actually let me foresee similar situations in the future…I have psychological preparation and more tools*.

*When I was the observer*,* I could sit and watch and think if I would do the same…what would I do if it’s my turn or what would happen in my emotion…I could pay more attention and compare different ways of doing.*

Moreover, the participants reflected on their own caregiving approach during debriefing after the simulation, in which they understood their actions and helped them apply in different contexts. In particular, the participants compared their own methods with those of others; then, they realized that they were doing well and reinforced themselves in caregiving. Furthermore, the participants appreciated interacting with the facilitator, who answered questions and engaged in conversation. Notably, the participants appreciated the feedback from the facilitator, which was reassuring and supportive.

*It [debriefing] lets us understand the technique used…then we would learn how to apply…for example*,* in this scenario it is about refusing to eat but in real life it could be refusing to join activities…I found that my own methods are not too bad compared to others…then it motivates myself…it’s like praising myself*,* encouraging myself that I am doing well.*

*The facilitator would answer my question*,* we would chat. The facilitator also confirms my approach as she commented and said: this works…It gives me reassurance…I feel supportive*.

#### Theme 3: perceived benefits

Through active learning, participants expressed benefits, including ‘enhanced skill in managing distressed behaviour and communication’, ‘enhanced caregiving knowledge’ and ‘reduced stress’, which became the three subthemes. The authentic conversation during the simulation deepened the participants’ memory of their skills in communication and managing distressed behaviour. Additionally, some participants expressed enhanced confidence in these two skills after roleplaying in the simulation. In particular, they highlighted learning how to react spontaneously to care recipients’ changing conditions.

*I took part in a scenario about handling repeating questions…when you get to experience*,* the memory is very strong…Now I am more confident in handling her repeated behavior…I would distract her by suggesting having snacks or making some white lies…In the scenario*,* I learnt that the condition keeps changing and I have to keep thinking of different solutions and spontaneously responding to these changing conditions*.

Moreover, the participants expressed enhanced caregiving knowledge, especially in the symptoms and reactions of care recipients. Through experience sharing, they also learnt various solutions for the same caregiving difficulty. Most importantly, during the learning process, they understood the importance of managing their own stress during caregiving and found ways to reduce it, including discussing their feelings and avoiding exaggeration.

*What they talked about is similar to my experience*,* and I understand that it’s not my mum pinpoint on me on purpose…these are the special signs and symptoms of dementia. Hearing others’ sharing*,* make me understand there are many other possible solutions and not just one…if my own method does not work then I can try the method I learn from other caregivers…so there are more ways out*.

*I learned how to relax myself…if I don’t relax myself*,* I would get depression…looking after him is harsh. I talked about my experience…it helps ventilate. I feel less stressed…learnt how to relax myself by not exaggerating the problem*.

#### Theme 4: implementation challenges

Negative feedback regarding simulation and home assignment was also noted, contributing to two subthemes: ‘mixed feelings about simulation’ and ‘incompletion of home assignment’. Most participants felt positive about the simulation, as it allowed them to share their approaches with other caregivers. However, some with less caregiving experience felt worried about the simulation due to concerns about their ability to react to the scenario, given their lack of similar experiences. In addition, participants found the scenario emotionally charged, which evoked sadness when they were immersed in it. Nevertheless, the participants suggested discussing possible solutions to the scenario among the caregivers beforehand, which may help prepare them psychologically. Moreover, some caregivers reported difficulty completing the home assignment because they had not encountered specific situations, such as fluctuating moods, as outlined in the assignment.

*I am worried that I cannot handle the scenario…there are other caregivers who are watching…I looked after my husband only in recent years…I am afraid that I could not think of any solutions or become mute. Also*,* I don’t want to be immersed in the character… those actors acted very well and made me feel real… I felt the stress*,* unhappiness and helpless being a caregiver in roleplaying in the scenario…if the facilitator could have talked about the scenario and gone through it before the roleplay… may provide more psychological preparation*.

*I may not be able to finish the assignment…for example… in the last week… my mother did not have any special emotion or nothing special happen*,* and she was very calm*,* so I am not able to finish the assignment*.

## Discussion

This feasibility study showed that the study design was feasible in the local community care settings, with a recruitment rate of 78%, an intervention completion rate of 93% and an attrition rate of 8.3%. Videoconferencing-based psychoeducation was also acceptable to the participants, as evidenced by the high satisfaction score and qualitative comments.

Despite the COVID-19 pandemic, the subject recruitment in this study was completed within a shorter time (5 months) than that in a similar study, which required 12 months to achieve a similar sample size [20]. The subject recruitment rate at 78% was higher than that reported in a similar study (64.7%), which compared psychoeducation in videoconference and in-person modes [22]. The current study adopted social media platform, Facebook, in addition to the community centers as the recruitment sources. The community center allowed its active member to access the study information through printed flyers; Facebook allowed the potential participants to remotely access the study information. Studies have reported that an increasing number of family caregivers of PLWD seek caregiving information and support through social media platforms such as Facebook and Twitter [43–45]. Therefore, the study information could have been exposed to many potential participants in a short time through Facebook. Nevertheless, several strategies have been adopted to enhance the exposure of study information to target participants on Facebook, including posting the study information in more than 5 Facebook forums that are specific to family caregivers of PLWD and reposting the study information every week.

Our findings demonstrated that the intervention design was acceptable to the participants in terms of its content and mode of delivery, as suggested by the high completion rate and satisfaction score. The completion rate was higher than the rates reported in previous studies, ranging from 74 to 89% [18, 20–22]. The difference is likely due to the adoption of simulation, group discussion and written assignment as the teaching and learning methods for active participation. Supported by the qualitative findings, active learning through active caregiver participation was highlighted as the key feature being appreciated by the participants. Previous studies have suggested that experiential learning or home assignment could effectively enhance the experience of participants in psychoeducational interventions [46, 47], as participants could actively engage in skill acquisition [48]. In addition, group discussions were reported to allow active learning from peers while sharing caregiving experiences [10, 11, 18, 48].

The participants valued the online format of psychoeducation, as they could remotely access the intervention. Similar findings were reported in previous studies in which online psychoeducation allowed family caregivers to access interventions when they could not leave their care recipient alone, had difficulties finding substitute care and had long travel times [18, 21, 49]. However, a special arrangement for the scheduling of online psychoeducation sessions was implemented to suit participants’ availability. As nearly half of the participants in the online group had full- or part-time work during the day, the online psychoeducation sessions were arranged in evenings during weekdays, especially for those who could not attend due to employment during the daytime. In addition to family caregivers, there are also migrant domestic workers providing round-the-clock domestic care for PLWD in some developed countries [49, 50]. Therefore, providing more options for the scheduling of online psychoeducation sessions to accommodate individuals’ schedules is suggested in future definite studies [18].

Moreover, the participants appreciated that videoconference facilitated open discussion of their own caregiving experiences, which was consistent with the qualitative findings reported in previous studies. Banbury et al. [51] reported that caregivers felt that it was easier to share their own caregiving experiences via videoconference at home than in-person, as they felt relaxed and less vulnerable in the home environment. The participants’ negative emotions, rooted in the high level of caregiving stress, were often expressed during the sharing of caregiving hardship. The relaxed and safe feelings provided by a familiar environment might ease their negative emotions and stress, thereby enhancing experience sharing. Therefore, videoconference at home could be employed as a possible way to facilitate discussion of caregiving experiences among Chinese family caregivers of PLWD, as the open sharing of emotion-laden experiences related to family problems is considered a cultural taboo in this population [19].

The retention of participants in the current study was greater than that reported in previous studies [18, 45]. In addition to the flexibility in scheduling sessions, sending reminders via WhatsApp one week and one day before the sessions could have contributed to the high retention. Besides, the importance of complete participation was explained to all participants. Furthermore, data collection was arranged at the participants’ convenience and was performed remotely.

Despite the considerable demographic differences between the intervention and control groups, their baseline levels of caregiving self-efficacy were comparable. Both groups demonstrated similarly high intervention satisfaction and completion rates. The participants in the intervention group were recruited via Facebook and were significantly younger, had higher education levels, and were adult children of PLWD. This finding may suggest that this subset of caregivers prefers to adopt online resources. Similar findings were reported from a survey that family caregivers of PLWD, who were younger and had attained higher education, were more likely to seek information online and were interested in online interventions related to dementia caregiving [52].

Both the online and in-person groups demonstrated significant within-group enhancement in caregiving self-efficacy post-intervention. However, no significant difference was demonstrated in the scores of the obtaining respite and controlling upsetting thoughts subscales between online and in-person psychoeducation. This finding contrasts with those of previous studies comparing videoconferencing group-based and in-person group-based psychoeducation, possibly because of the small sample size in the current study and the adoption of different tools for measuring caregiving self-efficacy [18, 22]. The nonsignificant difference in the scores of the obtaining respite and controlling upsetting thoughts subscales between the two groups may imply that videoconferencing group-based psychoeducation is at least comparable to in-person group- based psychoeducation in enhancing caregiving self-efficacy in obtaining respite and controlling upsetting thoughts of family caregivers of PLWD. Although a medium effect was demonstrated in enhancing caregiving self-efficacy in responding disturbed behaviours in the online group, it could be due to the higher education level of participants in the online group.

Literature suggests that family caregivers of PLWD reported suboptimal caregiving self-efficacy [4], which had a mediating role in physical and mental health (e.g., self-rated health, depressive symptoms, burden) [4, 53, 54]. These negative consequences could lead to neglect and avoidance in the family caregivers and thereby result in decreased quality of life in PLWD, frequent hospitalization, exacerbated BPSD or physical abuse [55]. On the contrary, enhancing the caregiving self-efficacy of family caregivers of PLWD may lead to lowered blood pressure and fatigue, and reduced caregiver burden and depressive symptoms [4, 56]. Therefore, it is paramount to deliver effective interventions for caregiving self-efficacy among the family caregivers of PLWD.

The virtual group meeting via videoconference simulates in-person group meetings and therefore allows synchronised interaction for feedback from interventionists, interactive coaching, and peer support [21], which are the cornerstones for caregiving self-efficacy [57]. This may have implication in clinical practice that videoconferencing group-based psychoeducation has the potential to complement in-person group-based psychoeducation in supporting the family caregiver who cannot attend in-person classes. This synchronised interaction is significant for family caregivers of PLWD in reducing social isolation and promoting peer support [58] when many caregivers have become socially isolated upon adopting their caregiving role [11]. Such peer support may reduce caregivers’ distress, mediated by caregiving self-efficacy [59], and in turn provide better quality of care.

The qualitative data suggested a mixed feeling towards simulation among the participants. The positive feelings included excitement, happiness, immersion, and authenticity. Similar findings were reported in a feasibility study in which simulated patients were employed to teach communication skills among family caregivers of patients diagnosed with head and neck cancer [59]. Mazanec et al. [59] reported that caregivers found simulation satisfying and helpful in learning communication skills, as the simulation was realistic and reflective of what was happening at home.

Nevertheless, the participants expressed negative feelings toward simulation, which highlighted the importance of the participants’ psychological preparation [60]. The anticipated worries may be due to insufficient knowledge and skills to avoid making mistakes in roleplay [61], which may jeopardize active engagement in learning. Therefore, providing a psychologically safe context for learning is crucial. In future trials, the noncompetitive nature of simulation should be explained to reduce anticipated worries. Additionally, the participants should acknowledge that mistakes may occur, which may be discussed in the context of debriefing. Furthermore, the interventionist should assist participants in achieving learning outcomes by going through the scenario beforehand and providing cues when situations deviate as planned [60].

There was neither caregiver discontinued participation due to technical difficulties nor technical issues regarding internet access and the use of the Zoom platform reported in this study. The interventionist attempted to minimize possible technical issues by offering a trial of logging on the Zoom platform before the first session, but it was refused by the participants. Instead, the interventionist logged into the Zoom platform 30 min before the start of every session so that the participants could test the audiovisual functions, which usually took no more than 5 min to complete. This may be because many family caregivers may have had experience attending other zooming activities during the COVID-19 pandemic when most in-person community support was suspended. Nevertheless, accessibility to online psychoeducation programs could be limited by socioeconomic status for internet access [21] and age, as globally 27% of those older than 65 years do not use the internet [62] and 38% of those older than 70 years do not use smartphones [63].

Given the confirmed feasibility and acceptability of videoconferencing-based psychoeducation, a definitive trial with a larger sample size is recommended to examine its effectiveness in enhancing the caregiving self-efficacy of family caregivers of PLWD. To successfully scale up the intervention implementation in a larger trial, involving multiple interventionists will be necessary. Healthcare professionals such as occupational therapists and social workers can be interventionists to promote active participation [19]. However, it is suggested that the interventionists receive simulation training to acquire the relevant skills and knowledge to facilitate simulation [64].

### Implications for practice and research

Our findings provide healthcare practitioners (e.g., nurses) and researchers with evidence to guide intervention planning for enhancing caregiving self-efficacy among family caregivers of PLWD. Notably, this study demonstrated that videoconferencing group-based psychoeducation is feasible, acceptable, and demonstrated preliminary effectiveness in enhancing the self-efficacy of responding to disruptive behaviours among Chinese family caregivers of PLWD. Healthcare practitioners may consider employing videoconferencing group-based psychoeducation to support those family caregivers who are socially isolated due to intense caregiving schedule or have barriers to attending in-person group-based psychoeducation. Future research should further examine the effectiveness of videoconferencing group-based psychoeducation with a stringent design and a larger sample size.

### Limitations

Several limitations in this study must be acknowledged. First, the satisfaction survey results could not reflect the acceptance of the booklet, as the perceived usefulness of the booklet was not measured specifically. Second, the study preliminary effect was subjected to bias and could be confounded by demographic characteristics, as significant differences in several demographic characteristics were demonstrated between groups. Group allocation without randomization could have led to this difference. Third, the preliminary effect was estimated on a small sample size; therefore, it may be underpowered to determine the true effect. Fourth, this study involved an active control group; therefore, it is impossible to determine if the preliminary effects were truly due to the intervention. As such, more studies are needed to explore whether videoconferencing group-based psychoeducation can complement or replace conventional in-person group-based psychoeducation. A stringent study design, such as a controlled trial design, with a larger sample size is warranted to examine the intervention effect. Fifth, given that a single interventionist carried out all the interventions in the current study, the study outcome, such as participants’ satisfaction, may be biased due to the interventionist’s particular personality, beliefs, and interactions with the participants, hence reducing the external validity and generalizability of findings [65]. Therefore, in the future, larger studies employing multiple interventionists should be considered, given that a standardised interventionist training is provided. Sixth, given that most of the participants in this study were female and had a relatively high level of education, the generalizability of the findings may be limited to a subset of caregivers who are female and highly educated. Seventh, long-term follow-up is lacking in this study because of resource implications. Caregiving self-efficacy may take time to emerge, particularly in the management of distressed behaviours, which are challenging [3]. It is suggested to have at least a follow-up 3 months post-intervention [66]. Finally, in addition to examining caregiving self-efficacy, future studies may also assess other outcomes, such as caregiver burden and caregiving stress to give a more comprehensive picture.

## Conclusion

This feasibility study provides evidence that videoconferencing group-based psychoeducation is feasible and acceptable for family caregivers of PLWD in the Chinese context. The recruitment rate (78%), overall attrition (8.3%), and participants’ satisfaction (4.7/5) were at an optimal level. Additionally, the qualitative findings revealed four themes: advantages of the online format, active learning, perceived benefits and implementation challenges. The participants appreciated the online format as handy and facilitated their open discussion of sensitive topics. They enjoyed the active learning (e.g., simulation and discussion), which enhanced their caregiving knowledge, skills, and emotional support. However, they expressed worries over failure in handling difficult communication and managing distressed behaviors during simulation training. Furthermore, there was a preliminary effectiveness in significantly enhancing the caregiving self-efficacy of responding to disruptive behaviours among the intervention group. Therefore, videoconferencing group-based psychoeducation may be comparable to in-person group-based psychoeducation in enhancing caregiving self-efficacy among the family caregivers of PLWD. As such, videoconferencing group-based psychoeducation has the potential to complement in-person group-based psychoeducation to support family caregivers who cannot attend in-person classes. A stringent study design, such as a randomised controlled trial design, of a larger sample size and long-term follow-up, is warranted to further examine the intervention effect.

## Supplementary Information

Supplementary Material 1.

## Data Availability

The datasets used and/or analysed during the current study are available from the corresponding author on reasonable request.

## Abbreviations

ADL: Activity daily living
BPSD: Behavioural and psychological symptoms of dementia
CONSORT: Consolidated Standards of Reporting Trials
CUT: Controlling upsetting thoughts
DECC: District Elderly Community Center
MoCA: Montreal Cognitive Assessment
OR: Obtaining Respite PLWD: people living with dementia
RDB: Responding to disruptive behaviors
RSCSE: Revised Scale of Caregiving Self-efficacy

## References

[1] Alzheimer’s Association. 2024 Alzheimer’s disease facts and figures. Alzheimer’s & Dementia. 2024;20:5:3708–3821. 10.1002/alz.13809 PMC11095490 38689398

[2] Steffen AM, Mc Kibbin C, Zeiss AM, Gallagher-Thompson D, Bandura A. The revised scale for caregiving self-efficacy reliability and validity studies. J Gerontol Ser B: Psychol Sci. 2002;57:74–86. 10.1093/geronb/57.1.p74 11773226

[3] Au A, Yip HM, Lai S, Ngai S, Cheng ST, Losada A, et al. Telephone-based behavioral activation intervention for dementia family caregivers: outcomes and mediation effect of a randomized controlled trial. Patient Educ Couns. 2019;102:2049–59. 31279613 10.1016/j.pec.2019.06.009

[4] Cheng ST, Li KK, Losada A, Zhang F, Au A, Thompson LW, et al. The effectiveness of nonpharmacological interventions for informal dementia caregivers: an updated systematic review and meta-anaylsis. Psychol Aging. 2020;35:55–77. 31985249 10.1037/pag0000401

[5] Leung DYP, Chan HYL, Chiu PKC, Lo RSK, Lee LLY. Source of social support and caregiving self-efficacy on caregiver burden and patient’s quality of life: a path analysis on patients with palliative care needs and their caregivers. Int J Environ Res Public Health. 2020;17:15. 10.3390/ijerph17155457 PMC7432213 32751147

[6] Dhabhar FS. Effects of stress on immune function: the good, the bad, and the beautiful. Immunol Res. 2014;58:193–210. 24798553 10.1007/s12026-014-8517-0

[7] World Health Organization. iSupport. 2024. https://www.who.int/teams/mental-health-and-substance-use/treatment-care/isupport. Accessed 1 Aug 2023.

[8] Smith GD, Ho K, Lee A, Lam L, Chan C. Dementia literacy in an ageing world. J Adv Nurs. 2022;79:6:2167–74. 36582068 10.1111/jan.15556

[9] Kales HC, Gitlin LN, Stanislawski B, Marx K, Turnwald M, Watkins DC, et al. WeCareAdvisortm: the development of a caregiver-focused, web-based program to assess and manage behavioral and psychological symptoms of dementia. Alzheimer Disease Assoc Diorders. 2016;31:3. 10.1097/WAD.0000000000000177 PMC5432421 27849639

[10] Samia LW, O’Sullivan A, Fallon KC, Aboueissa AM, Hepburn KW. Building on self-efficacy for experienced family caregivers: the savvy advanced program. Gerontologist. 2019;59:5. 10.1093/geront/gny016 29546325

[11] Sepe-Monti M, Vanacore N, Bartorelli L, Tognetti A, Giubilei F, Savvy Caregiver Study Group. The savvy caregiver program: A probe multicenter randomized controlled pilot trial in caregivers of patients affected by alzheimer’s disease. J Alzheimer’s Disease. 2016;54:1235–46. 27567824 10.3233/JAD-160235

[12] Csipke E, Shafayat A, Sprange K, Bradshaw L, Montgomery AA, Ogollah R, et al. Promoting independence in dementia (PRIDE): a feasibility randomized controlled trial. Clin Interv Aging. 2021;16:363–78. 33664568 10.2147/CIA.S281139 PMC7921631

[13] Cuffaro L, Di Lorenzo F, Bonavita S, Tedeschi G, Leocani L, Lavorgna L. Dementia care and COVID-19 pandemic: A necessary digital revolution. Neurol Sci. 2020;41:8. 10.1007/s10072-020-04512-4 PMC7298162 32556746

[14] Frias CE, Garcia-Pascual M, Montoro M, Ribas N, Risco E, Zabalegui A. Effectiveness of a psychoeducational intervention for caregivers of people with dementia with regard to burden, anxiety and depression: a systematic review. J Adv Nurs. 2020;76:787–802. 31808211 10.1111/jan.14286

[15] Walter E, Pinquart M. How effective are dementia caregiver interventions? An updated comprehensive meta-analysis. Gerontologist. 2020;60:8. 10.1093/geront/gnz118 33226434

[16] Bandura A, Self-Efficacy. The exercise of control. New York: W. H. Freeman and Company; 1997.

[17] Parra-Vidales E, Soto-Perez F, Perea-Bartolome MV, Franco-Martin MA, Munoz-Sanchez JL. Online interventions for caregivers of people with dementia: A systematic review. Actas Esp Psiquiatr. 2017;45:3. 28594057

[18] Hepburn K, Nocera J, Higgins M, Epps F, Brewster GS, Lindauer A, et al. Results of a randomized trial testing the efficacy of Tele-Savvy, an online synchronous/asynchronous psychoeducation program for family caregivers of persons living with dementia. Gerontologist. 2021. 10.1093/geront/gnab029. 10.1093/geront/gnab029 PMC7989248 33640979

[19] Chan JHM, Ho KHM, Pang RCK, Chan YL. Strategies and factors to enhance active participation of family caregivers of people with dementia in psychoeducation: a scoping review. Dementia. 2023;23:2. 10.1177/14713012231220231 38091474

[20] Griffiths PC, Kovaleva M, Higgins M, Langston AH, Hepburn K. Tele-Savvy: an online program for dementia caregivers. Am J Alzheimer’s Disease Other Dement. 2018;33:5. 10.1177/1533317518755331 PMC10852441 29544342

[21] Karagiozi K, Margaritidou P, Tsatali M, Marina M, Dimitriou T, Apostolidis H, et al. Comparison of on site versus online psychoeducation groups and reducing caregiver burden. Clin Gerontologist. 2022;45(5):1330–40. 10.1080/07317115.2021.1940409 34219617

[22] Noel MA, Lackey E, Labi V, Bouldin ED. Efficacy of a virtual education program for family caregivers of persons with living with dementia. J Alzheimer’s Disease. 2022;86:1667–78. 35213371 10.3233/JAD-215359 PMC9108574

[23] Bond C, Lancaster GA, Campbell M, Chan C, Eddy S, Hopewell S, et al. Pilot and feasibility studies: extending the conceptual framework. Pilot Feasibility Stud. 2023;9:24. 36759879 10.1186/s40814-023-01233-1 PMC9909985

[24] Griffiths PC, Whitney MK, Kovaleva M, Hepburn K. Development and implementation of Tele-Savvy for dementia caregivers: a development of veterans affairs clinical demonstration project. Gerontologist. 2016;56:1. 26566806 10.1093/geront/gnv123

[25] Census and Statistics Department. Thematic Household Survey Report No. 75: Internet and personal computer penetration. 2022. http://www.censtatd.gov.hk. Accessed 1 Aug 2023.

[26] Oh SS, Kim KA, Kim M, Oh J, Chu SH, Choi JY. Measurement of digital literacy among older adults: systematic review. J Med Internet Res. 2021;23:2e26145. 10.2196/26145 PMC7889415 33533727

[27] Kane T, Hammad SH, Islam N, Al-Wattary N, Clark J, Daher-Nashif S. Dementia caregiving in the middle East and North africa: A scoping review. Transcult Psychiatry. 2021;58(6):844–58. 10.1177/13634615211036404. 34407707 10.1177/13634615211036404

[28] Eldridge SM, Chan CL, Campbell MJ, Bond CM, Hopewell S, Thabane L, et al. CONSORT 2010 statement: extension to randomized pilot and feasibility trials. BMJ. 2016;355:i5239. 27777223 10.1136/bmj.i5239 PMC5076380

[29] Folstein MF, Folstein SE, McHugh PR. Mini-mental state. A practical method for grading the cognitive state of patients for the clinician. J Psychiatr Res. 1975;12:189–98. 1202204 10.1016/0022-3956(75)90026-6

[30] Fortinsky RH, Kercher K, Burant CJ. Measurement and correlates of family caregiver self-efficacy for managing dementia. Aging Ment Health. 2002;6:2. 10.1080/13607860220126763 12028884

[31] Gonyea JG, Lopez LM, Velasquez EH. The effectiveness of a culturally sensitive cognitive behavioral group intervention for Latino alzheimer’s caregivers. Gerontologist. 2014;56:292–302. 24855313 10.1093/geront/gnu045 PMC4906638

[32] Pearlin LI, Mullan JT, Semple SJ, Skaff MM. Caregiving and the stress process: an overview of concepts and their measures. Gerontologist. 1990;30:5. 2276631 10.1093/geront/30.5.583

[33] Browne RH. On the use of a pilot sample for sample size determination. Stat Med. 1995;14:1933–40. 8532986 10.1002/sim.4780141709

[34] Kolb DA. Experiential learning: experience as the source of learning and development. New Jersey: Prentice Hall; 1984.

[35] Yau KKY, Loke AY. Effects of diaphragmatic deep breathing exercises on prehypertensive or hypertensive adults: A literature review. Complement Ther Clin Pract. 2021;43:101315. 33530033 10.1016/j.ctcp.2021.101315

[36] Yeung CCY, Ho KHM, Chan HYL. A dyadic advance care planning intervention for people with early-stage dementia and their family caregivers in a community care setting: a feasibility trial. BMC Geriatr. 2023;23:115. 36859250 10.1186/s12877-023-03815-3 PMC9979490

[37] Au A, Lai MK, Lau KM, Pan PC, Lam L, Thompson L, et al. Social support and well-being of dementia family caregivers: the mediating role of self-efficacy. Aging Ment Health. 2009;13:5. 10.1080/13607860902918223 19882415

[38] Pallant J. SPSS survival manual: A step by step guide to data analysis using IBM SPSS. 7th ed. New York: Routledge; 2020.

[39] Cohen J. Statistical power analysis for the behavioual sciences. 2nd ed. New York: Routledge; 1988.

[40] Elo S, Kyngas H. The qualitative content analysis process. J Adv Nurs. 2007;62:107–15. 10.1111/j.1365-2648.2007.04569.x 18352969

[41] Lincoln YS, Guba EG. Naturalistic inquiry. Sage; 1985.

[42] Chen HY, Boore JR. Translation and backtranslation in qualitative nursing research: methodological review. J Clin Nurs. 2010;19(1–2):234–9. 19886874 10.1111/j.1365-2702.2009.02896.x

[43] Lo YC. Social media use and empowerment in dementia care: Facebook groups for family caregivers as an example. J Nurs. 2023;70:2. 10.6224/JN.202304_70(2).04 36942539

[44] Mehta NL, Zhu L, Lam K, Stall NM, Savage R, Read SH, et al. Health forums and Twitter for dementia research: opportunities and considerations. J Am Geriatr Soc. 2020;68:12. 10.1111/jgs.16790 32894780

[45] Teles S, Paul C, Costa-Santos C, Ferreira A. Use of dementia and caregiving-related internet resources by informal caregivers: a cross-sectional study. Front Med. 2022;9978635. 10.3389/fmed.2022.978635. 10.3389/fmed.2022.978635 PMC9518752 36186787

[46] Llanque SM, Enriquez M, Cheng AL, Doty L, Brotto MA, Kelly PJ, et al. The family series workshop: a community-based psychoeducational intervention. Am J Alzheimer’s Disease Other Dementias. 2015;30:573–83. 10.1177/1533317514568003 PMC4508226 25609602

[47] Sorensen S, Pinquart M, Duberstein P. How effective are interventions with caregivers? An updated meta-analysis. Gerontologist. 2002;42:3. 10.1093/geront/42.3.356 12040138

[48] Chen HM, Huang MF, Yeh YC, Huang WH, Chen CS. Effectiveness of coping strategies intervention on caregiver burden among caregivers of elderly patients with dementia. Psychogeriatrics. 2015;15:20–5. 25515800 10.1111/psyg.12071

[49] Boots LMM, de Vugt ME, Kempen GIJM, Verhey FRJ. Effectiveness of a blended care self-management program for caregivers of people with early-stage dementia (Partner in Balance): randomized controlled trial. J Med Internet Res. 2018;20:7. 10.2196/10017 PMC6064039 30006327

[50] Ho KHM, Yang C, Leung AKY, Bressington D, Chien WT, Cheng Q, et al. Peer support and mental health of migrant domestic workers: a scoping review. Int J Environ Res Public Health. 2022;19:137617. 10.3390/ijerph19137617 PMC9265321 35805278

[51] Banbury A, Parkinson L, Gordon S, Wood D. Implementing a peer-support programme by group videoconferencing for isolated carers of people with dementia. J Telemed Telecare. 2019;25:9. 10.1177/1357633X19873793 31631761

[52] Ho DWH, Mak V, Kwok TCY, Au A, Ho FKY. Development of a web-based training program for dementia caregivers in Hong Kong. Clin Gerontologist. 2015;38:3.

[53] Wawrziczny E, Berna G, Ducharme F, Kergoat M-J, Pasquier F, Antoine P. Modeling the distress of spousal caregivers of people with dementia. J Alzheimer’s Disease. 2016;55(2):703–16. 10.3233/JAD-160558. 10.3233/JAD-160558 27716667

[54] Wawrziczny E, Antoine P, Doba K. Modeling the Distress of Adult-Child Caregivers of People with Dementia: The Mediating Role of Self-Efficacy. Journal of Alzheimer’s Disease. 2021;84(2):855–867. 10.3233/JAD-210624. PMID: 34602477. 10.3233/JAD-210624 34602477

[55] Stall NM, Kim SJ, Hardacre KA, Shah PS, Straus SE, Bronskill SE, Lix LM, Bell CM, Rochonet PA. Association of informal caregiver distress with health outcomes of community-dwelling dementia care recipients: A systematic review. J Am Geriatric Soc. 2019;67(3):609–17. 10.1111/jgs.15690 30536383

[56] Crelilin NE, Orell M, McDemott O, Charlesworth G. Self-efficacy and health related quality of life in family carers of people with dementia: A systematic review. Aging Mental Health. 2014;18(8):954–69. 24943873 10.1080/13607863.2014.915921 PMC4192898

[57] Khan TS, Hirschman KB, McHugh MD, Naylor MD. Self-efficacy for family caregivers of older adults with cognitive impairment: A concept analysis. Nurs Forum. 2021;56:1. 10.1111/nuf.12499 PMC8549654 32888197

[58] Seike A, Sumigaki C, Takeuchi S, Hagihara J, Takeda A, Becker C, et al. Efficacy of group-based multi-component psycho-education for caregivers of people with dementia: A randomized controlled study. Geriatr Gerontol Int. 2021;21:561–7. 33949065 10.1111/ggi.14175

[59] Mazanec SR, Sandstrom K, Coletta D, Dorth J, Zender C, Alfes CM, et al. Building family caregiver skills using a simulation-based intervention: A randomized pilot trial. Oncol Nurs Forum. 2019;46:4. 10.1188/19.ONF.419-427 31225839

[60] Committee INACSLS, Watts PI, McDermott DS, Alinier G, Charnetski M, Nawathe PA. Healthcare simulation standards of best Practice™ simulation design. Clin Simul Nurs. 2021;58:14–21.

[61] Rudolph J, Raemer DB, Simon R. Establishing a safe container for learning in simulation: the role of the presimulation briefing. Simul Healthcare: J Soc Simul Healthc. 2014;9:6:339–49. 10.1097/SIH.0000000000000047 25188485

[62] Pew Research Center. Share of adults in the United States who use the internet in 2019, by age group. 2019. http://www.statista.com/statistics/266587/percentage-of-internet-users-by-age-group-in-the-us. Accessed 28 Aug 2023.

[63] Kakulla NB. Tech trends of the 50+. AARP Res. 2020. 10.26419/res.00329.001.

[64] Standards Committee INACSL, Persico L, Belle A, DiGregorio H, Wilson-Keates B, Shelton C. Healthcare simulation standards of best Practice™ facilitation. Clin Simul Nurs. 2021;58:22–6.

[65] Goto Y, Miura H. An exploratory study of issues in training facilitators for online training in advance care planning: mixed methods research. Nurs Rep. 2024;14(2):1000–13. 10.3390/nursrep14020075. 38651487 10.3390/nursrep14020075 PMC11036261

[66] Cristancho-Lacroix V, Wrobel J, Cantegriel-Kallen I, Dub T, Rouquette A, Rigaud AS. A web-based psychoeducational program for informal caregivers of patients with alzheimer’s disease: A pilot randomized controlled trial. J Med Internet Res. 2015;17:5e117. 10.2196/jmir.3717 PMC4468784 25967983

